# Family planning policy and gender in Nigeria: A thematic analysis of the government's health policy perspective

**DOI:** 10.1111/1467-9566.13853

**Published:** 2024-10-01

**Authors:** Obreniokibo Ibifubara Amiesimaka, Shahin Payam

**Affiliations:** ^1^ Chair of Sociology of Diversity Department of Sport and Health Sciences Technical University of Munich Munich Germany; ^2^ Department of Preventive and Social Medicine University of Otago Dunedin New Zealand; ^3^ Psychology School Hochschule Fresenius—University of Applied Sciences Munich Germany

**Keywords:** family planning, gender, Nigeria, policy, thematic analysis

## Abstract

Family Planning (FP) lets people control the number and timing of child‐births through using contraceptives and/or restoring fertility. Nigeria has several FP policies for managing its population, yet contraceptives usage remains suboptimal despite high FP awareness, suggesting that several factors might be inhibiting FP uptake. The literature spotlights gender as factoring into FP use due to Nigeria’s patriarchal society, with men positioned as gatekeepers to women’s sexual/reproductive health/expression. Therefore, we investigate if/how gender is considered in Nigeria’s FP policies. We thematically analysed the ‘National Reproductive Health Policy’, ‘National Family Planning Communication Plan’ and ‘Nigeria Family Planning Blueprint (Scale‐Up Plan)’, from a critical realist viewpoint. Our analysis generated an overarching theme—‘A Gendered Human Right’, with three further themes: Women’s Right—Women’s Issue’, ‘Adolescent Girls—not left out’ and ‘Men’s Right as Supporters’. FP was portrayed as the right of women, adolescents (particularly girls ≥ 10 yrs) and men. It was highly feminised, with women, not men, majorly shouldering the FP responsibility and women’s FP access was presented as hindered by men. Moreover, we advance recommendations for optimising Nigeria’s policies to address gender imbalances hampering women’s FP access and uphold the rights of all people, women/girls especially.

## INTRODUCTION

Nigeria is ranked sixth by population globally and is forecast to rise to third by 2050 (UN Data, 2023). Women in the country each have approximately five children, supporting the population of 206 million, which is increasing yearly at a rate of 2.6% (UNFPA, 2020). Such exponential population growth raises concerns about its sustainability given the strain on public services and the environment, coupled with other development issues that arise as a result (Visagie & Turok, 2020, National Population Commission (NPC), 2021).

Family planning (FP) is a means of controlling the number of children one has, as well as the time interval between their births; this is accomplished mainly with the use of contraceptive methods besides through treating infertility (WHO, 2023a). Prevention of pregnancy by contraceptives lasts different durations, with hormonal methods such as “the pill” or barrier methods like condoms and the diaphragm/cap, providing short term protection (WHO, 2023b). Long‐acting reversible contraceptives (LARCs) are majorly hormonal methods (containing ovulation‐interrupting hormones, for example oestrogen and progestogen, etc.), such as injectables, implants and intrauterine devices (IUDs), though non‐hormonal (i.e. copper) IUDs also exist. FP methods like vasectomy and tubal ligation for men and women, respectively, can be used to achieve permanent protection (although special procedures exist to reverse both of these contraceptive methods) (WHO, 2023a; WHO, 2023b). All these are ‘modern’ methods of contraception, which involve medical interventions; conversely, there are also ‘natural’ methods which do not.

Natural methods, such as the standard days, temperature and cervical secretion monitoring methods, require a woman to keep track of her menstrual cycle and avoid unprotected intercourse during her most fertile days chiefly by utilising barrier methods (NHS, 2024; Schwarz et al., 2021).

FP has been touted as instrumental in slowing the population growth rate (Liu & Raftery, 2020) and as a “silver bullet” for mitigating a host of social challenges. Again, its merits include positive effects on the economy, public health, education, sustainable population management and even on climate change (Rimon & Tsui, 2018; WHO, 2023a).

Only 20.1% of Nigerian women of reproductive age (WRA) use any contraceptives, whilst 19% of the need for contraceptives is yet unmet (UN, 2023). This falls considerably short of the contraceptive prevalence rate (CPR) target of 36% by 2018 the Nigerian government set for itself (Federal Ministry of Health (FMoH), 2017a). Incidentally, FP awareness is quite high, as 83.8% of WRA know about modern FP methods (FMoH, 2017a); notwithstanding, just 15% use them, with another 5.1% employing traditional methods (UN, 2023). This represents a knowledge—uptake gap, needing proper assessment.

FP is a fundamental and inextricable component of health, evidenced by its inclusion in the Sustainable Development Goals (SDG) 3.7 and 5.6 on sexual and reproductive health (SRH) (Starrs et al., 2018; UN Statistics Division, 2023). Therefore, in order to close the aforementioned knowledge—uptake gap, the Federal Ministry of Health (FMoH) launched the revised National Reproductive Health Policy (NRHP) in 2017 (FMoH, 2017b). This document is complemented by the National Family Planning Communication Plan (NFPCP), itself the offshoot of the 2014 Nigeria Family Planning Blueprint (Scale‐Up Plan) (NFPB) (FMoH, 2014; FMoH, 2017a).

Amiesimaka and Payam (2020) point out that gender plays a material role in the perception and uptake of FP methods in Nigeria. The authors highlight that Nigeria is culturally patriarchal, a situation underscored by conservative, traditional and religious, views as well as patrilineal heritage. Thus, the power dynamic is skewed greatly in favour of men, allowing them be the ultimate decision makers for a variety of issues including on women’s (their wives or female partners) sexual and reproductive health. Decisions about how many children a woman has and how frequently they are born are substantially influenced by the man’s heritage family (e.g. his parents) (Ajala et al., 2022; Aransiola et al., 2014). Buttressing this, contraceptive use is substantially hampered in homes with live‐in in‐laws (Fadeyibi et al., 2022).

Despite many initiatives and laws in furtherance of gender equality in Nigeria, Nigeria still places at 130 among 146 countries in the Global Gender Gap Report 2023 (World Economic Forum (WEF), 2023). The extent of gender inequality is important for FP as studies have found that better empowered women—financially, educationally, socially, etc.—are more likely to use FP methods (Corroon et al., 2014; Duru et al., 2018; Oluwasanu et al., 2019).

Furthermore, as FP is mainly framed within the context of (heterosexual) coupledom, it is imperative to consider if/how the government takes cognisance of gender dynamics in its family planning policies as they might underpin the limited FP uptake. To this end, we conduct a reflexive thematic analysis of the NRHP, the NFPCP and the NFPB in this article. This is in line with the recommendation of Amiesimaka and Payam (2020), where the authors, drawing upon the literature, outline the gender dynamics in Nigeria in relation to FP. They posit that men are presented as in charge of FP decisions, supported by a conservative culture and religious bent as well as insufficient women’s empowerment. On this background, the authors suggest that due consideration be given to how gender is considered in Nigerian FP policies. The in‐depth view of the government’s perception of gender in FP promotion obtained therefrom would be useful in understanding the Nigerian contextualisation of FP, as well as for providing insights for the development of FP promotion strategies.

## RESEARCH METHODS

### Theoretical framework

This research is broadly situated within a critical realist framework, with the analysis approached from a feminist perspective (Gunnarsson et al., 2016). Critical realism is a mid‐point position on the epistemology/ontology continuum, which holds that there is indeed a “reality” but concedes that reality is not directly accessible to a researcher (Burr, 2015; Willig, 2013). This is because “reality” is filtered through the prism of socio‐cultural factors, including language, culture, etc. and ‘real’ structures/objects are evident often only through their effects (Burr, 2015; Clarke & Braun, 2013a). “Critical realism is ‘critical’ because it tries to uncover the implicit and potentially misleading or damaging assumptions of various social policies and ways of thinking; it is interested in generating knowledge that is capable of working in the best interests of people” (Burr, 2015). Hence, the data were approached with “suspicion” and not taken at “face value”, but the structures which undergird the perception of the reality it suggests were assessed (Burr, 2015; Willig, 2013).

Central to critical realism is the separation of ontology (being and doing) from epistemology (thinking) (Alderson, 2021a) and this research adopts a realist ontology with a social constructionist epistemology. To this end, we took cognisance of the fact that our personal experiences and viewpoints, captured in the reflexivity section below, coloured our thinking in identifying, analysing and presenting the data—using methods outlayed in the sections below. Given critical realisms’ recognition of injustices and inequalities in society alongside its “concern with holistic social progress” (Alderson, 2021b), we highlight that we conducted this research using a feminist lens, holding steadfast to our conviction that women and men are equal in all respects and women should have full control over their bodies, reproduction and sexuality. This imbricated feminism and critical realism nexus is well‐supported by the literature (Gunnarsson et al., 2016).

### Documentation analysis (DA)

We adopted the research method of documentation analysis (DA) in conducting this study as we focussed on the Nigerian government’s view of family planning (FP) reflected in its policy documents publicly available on the Federal Ministry of Health (FMoH) website. DA is “a systematic procedure for reviewing or evaluating documents” which entails a preliminary read through and then a detailed perusal of the document(s), with data interpretation following these (Bowen, 2009). Hence, we were able to circumvent socially desirable responses due to the interactive setting in other data collection methods (e.g. interviews, focus group discussions), while gaining a holistic perspective of FP through looking at the published documents (Sommerhoff et al., 2018). Nonetheless, we note that government documents such as the ones we analyse inherently reflect the perspectives of the authors which would inevitably impact our research.

#### Reflexive thematic analysis (RTA)

As DA must be complemented by an appropriate evaluation technique to generate the meanings in the data (Corbin & Strauss, 2014), we analysed the documents using reflexive thematic analysis (RTA) as prescribed by Braun and Clarke (2006), Braun et al. (2018), Braun and Clarke (2019). These authors define RTA as “a method for identifying, analysing and reporting patterns (themes) within data” (Braun & Clarke, 2006). These themes are “patterns” which indicate unifying meaning, grounded in a fundamental concept (Braun et al., 2014, 2018). We worked inductively—putting the data at the forefront of our analysis—and also worked on both a semantic and latent level—including surface‐level themes describing the data, alongside interpretive themes going deeper than the plain meanings of the data (Campbell et al., 2021).

Essentially, themes generated through RTA underscore salient points across the data, whilst presenting them in a detailed, yet condensed manner. RTA is very versatile as it can be applied to data obtained through different means, including texts (e.g. documents, reports, diaries), focus group discussions, interviews, etc (Braun et al., 2018; Clarke & Braun, 2013b). Again, RTA lends itself to the diversity of epistemological standpoints; spanning the range from essentialism to constructionism. This is as RTA focusses on patterns at the level of language, which are fairly removed from the theoretical framework (Clarke & Braun, 2013b). Hence, RTA is an effective tool for investigating elements that undergird and/or situate phenomena in context (Braun et al., 2018).

We adopted RTA, as it is fully undergirded by the qualitative paradigm (Braun et al., 2018). This approach firmly maintains that context is essential to meaning, various realities exist and the subjectivity of researchers is a “resource” to be harnessed (Clarke & Braun, 2013a). Hence, this study’s themes were actively “generated” by the researchers via an open coding analysis process and not “revealed” through the analysis (Braun & Clarke, 2016; Braun et al., 2018). Moreover, Clarke and Braun (2013b) advocate for thematic analysis to be conducted in six steps; these were followed in this study. First, OIA familiarised himself with the data through perusal. Second, OIA underscored the basic relevant details in the data through the coding process. Third, OIA developed themes by grouping related codes. Next, OIA reviewed the themes for congruence with the dataset. Sequel to this, OIA defined the themes via robust naming and expounding. An iterative process followed whereby SP reviewed these five steps until the generated themes were refined and robust. The composition of this paper constituted the final step as it serves to paint a comprehensive picture of the data using the analysed themes. It was championed by OIA with the review of SP.

#### Data collection

We analysed the following documents: the National Reproductive Health Policy 2017 (NRHP), the National Family Planning Communication Plan 2017–2020 (NFPCP) and the Nigeria Family Planning Blueprint (Scale‐Up Plan) (NFPB) of 2014. The NFPCP’s purpose is to detail the strategy for an “aggressive” FP communication campaign to surmount the barriers hampering the use of FP services/methods (FMoH, 2017a). Consequently, its target audience are all stakeholders in FP, including those in the private, NGO, civil society and public (national, state and local) sectors, alongside the general public.

Developing the NFPCP was one of the outcomes of the NFPB, which was devised as an all‐encompassing strategy to promote FP use via multiple interventions. Again, the NRHP seeks to provide an outlay of the reproductive health sphere (unequivocally including FP), set out a new direction for the field and prescribe roles for relevant players (FMoH, 2017b). Its target audience is, hence, all reproductive health stakeholders across diverse fields together with the public.

As FP is a central component of sexual and reproductive health (SRH) (Starrs et al., 2018; UNSD, 2023), the Nigerian FMoH plays a central role in country‐wide FP policy conceptualisation, facilitation and service provision. Therefore, we focussed on these documents authored and implemented by the FMoH, Nigeria’s highest level of health authority. Nonetheless, we note that other government agencies have launched policies related to FP, particularly the National Policy on Population for Sustainable Development (NPPSD) (NPC, 2021). However, this policy does not formally build upon the FMoH policies and only sought to update the 2004 NPPSD in light of documents including: Agenda 2063 for African Development, the Nigeria Economic Recovery and Growth Plan (2017–2020), the Paris Agreement on Climate Change (2015), amongst others. Since this study entailed thematic analysis of publicly available documents, specific ethics committee approval was not required.

### Reflexivity

Reflexivity is essential in qualitative research for interrogating the ways through which a researcher might impact the research outcomes, as it is not possible for a researcher to stay completely dispassionate with regard to their research. Reflexivity allows for introspection on how the researcher engages in the “construction of meanings” during the research (Willig, 2013).

We, OIA and SP, both have lived in several Western countries, have had very liberal upbringings, come from a social justice perspective, adopt feminist approaches and have one sibling—a sister—each. Further, we both have migrant backgrounds as SP is German‐born with multi‐cultural Eastern migrant parents and OIA is Nigerian by birth. SP has worked on tackling gender inequality and ethical work with children; whilst OIA has worked in family planning promotion and policy research in Nigeria, gaining first‐hand knowledge of the country’s socio‐cultural nuances regarding FP. Consequently, we support FP promotion and believe that besides empowering women to have control over their own bodies, FP helps every person have just as many children as they desire and can provide for, while the country is able to avoid the socio‐economic challenges of over‐population.

However, neither of us are cis‐women, with SP identifying as a man and OIA also identifying as a man, but is not constrained by gender norms. We acknowledge that FP and contraceptives are often couched as pertaining mainly to women and also that we lack the experience or possibility of being pregnant. Nonetheless, our gender identities afford us distinct, non‐mainstream, perspectives on the topic as we are able to view the data *from a distance* by virtue of being uninvolved in the conventional settings of FP discourse. Such “outsider” standpoints have unique value for qualitative analyses (Berkowitz et al., 2007; Schmid et al., 2023).

It is these experiences that inspired our decision to conduct this research and how we executed this project.

## FINDINGS

An overarching motif for this thematic analysis was the couching of FP as an intrinsic component of sexual and reproductive health (SRH). This informed the coding of explicit mentions of SRH, particularly in the NRHP, as relating indubitably to FP. Three themes about gender in relation to family planning (FP) were generated within the concept of human rights as represented by the overarching theme—“a Gendered Human Right”. The themes—“Women’s Right—Women’s Issue”, “Adolescent Girls—not left out” and “Men’s Right as Supporters”—provide deeper insight into the nuances of gender and FP.

### A Gendered Human Right

Across the three documents, FP was framed as a right, albeit gendered. This characterisation of FP as a human right reflects the Nigerian government’s view that it is not a privilege that can be bestowed discretionarily, but an entitlement that must be honoured. The quote below illustrates this:

“…this policy recognises the right of all persons to the highest attainable standard of health, particularly as regards their reproductive and sexual health including the rights of individuals and couples to access relevant reproductive health information and quality services…”

(NRHP p. 19)

Nigeria frames FP, alongside SRH, as a right of the people and further portrays this right as pertaining to FP information and service access. Nigeria thus situated these two factors as essential for the enjoyment of the right. This rights‐based formulation obviates the need for any justification for promoting, funding or providing FP services on the part of the government and provides grounds for individuals to demand for such services.

More so, this conceptualisation of FP as a human right is also couched as providing liberation and choice, enabling the individual to take control of decisions relating to their fertility and procreation. This is expanded upon in the next quote:

“Reproductive health therefore implies that people are able to have a satisfying and safe sex life and that they have the capability to reproduce and the freedom to decide if, when and how often to do so”

(NRHP p. 1)

Also included in this concept of FP as sexual liberation, is the right to a “satisfying and safe sex life”. This aligns with feminist views of women’s sexuality and contraception, which hold that FP facilitates female sexual expression by allowing for the control of pregnancy as an outcome (Lowe, 2005; Wigginton et al., 2015). This sexual liberation formulation also encourages people not using FP within the context of families (e.g. the unmarried) to access contraception in exercise of their right to reproductive health. This is important as, by its very name, family planning connotes a context of raising a family and thus could be perceived as exclusionary.

The concept of choice in FP as a right also relates to the opportunity to decide which FP method to use; hence, FP service providers are:

“Requested to provide information on the various modern Family Planning methods so that clients can make informed choices on the most appropriate methods for them”

(NFPCP p. 12)

The right to FP is depicted as including the freedom to choose between the diverse FP methods, albeit limited to modern ones, suggesting a deprioritisation of natural methods. To enable this decision‐making process, Nigeria makes provision for adequate information to be made available to empower prospective users in enjoying that freedom. Presumably, the variety of FP methods themselves would also be provided to give users options to choose from.

Moreover, FP as a human right is framed as universal; the following excerpt illustrates this:

“All Nigerians, irrespective of their gender and age including adolescents from age 10 years and older population, have sexual and reproductive rights and are equally entitled to sexual and reproductive health development and care.”

(NRHP p. 19)

Nigeria frames the right to FP as extended to all people; be they men, women or adolescents. This constitutes a basis for the government to provide FP services in a non‐discriminatory manner to (prospective) users.

In characterising FP as a human right, Nigeria provides a seemingly incontestable basis for people to have unfettered access to FP. This rights‐based take on FP is in tandem with the prevailing view of FP in the health, as well as socio‐psychological community, which holds that FP enables the right of individuals to control their fertility patterns (Raday et al., 2017; Sinai et al., 2020). Although Nigeria frames these rights as applying to all individuals in a non‐discriminatory manner, there is a gendered aspect apparent, as outlined next.

#### Women’s Right—Women’s issue

Within the context of the right to FP, the needs of women are specially spotlighted by the Nigerian government. It positions women at the centre of home building within family structures, as is typical in patriarchal societies (Abdi et al., 2021; Sikweyiya et al., 2020). This theme explores the diverse ways, both direct and indirect, that FP is presented as particularly important for women to enjoy their human rights. This is illustrated below:

“Implicit in this last condition are the right of …women to be informed and to have access to safe, effective, affordable and acceptable methods of family planning of their choice, as well as other methods of their choice for the regulation of fertility which are not against the law”

(NRHP p. 1)

FP as a right is framed as enabling women to exercise their free choice and control over their fecundity. Noteworthy is that the government does not say that women have a right to “inform themselves” about FP methods, but instead says that women have a right to “be informed” about them. The implication of this is that another entity—the government—has to provide the avenue for its redemption. Likewise, in order to “have access to …FP”, the FP methods must be placed at the disposal of women. Women are thus positioned as more passive with the government taking the lead in facilitating the FP process. Furthermore, this framing of FP as a women’s right is characterised as being limited by legality; thus suggesting that this right has the backing of the law, because what is permitted must be clearly defined so that what is illegal can be delineated.

The government’s specific targeting of women is apparent in the following extract:

“The intended primary audiences are an approximated 7.3 million women who say they do not wish to get pregnant now or ever again and who are not using a Family Planning method resulting in unmet need.”

(NFPCP p. 10)

Here, Nigeria presents FP as a women’s issue and indicates that its efforts are geared towards facilitating the exercise of the women’s right to FP. Further, by focussing on women with an unmet need, Nigeria gauges the willingness of women to use FP. Hence, the government presents FP as something women have signified a *need* for even though they might not have expressly indicated that they *want* FP (unmet need). This characterises FP as fulfiling the desires of women who already, of their own accord, have indicated interest in reproductive outcomes that FP can deliver. This formulation serves to justify the programmatic interventions made in FP promotion.

Still on FP as a women’s issue, the policies further say:

“Knowledge and demand will come from …the encouragement of FP use to promote the health of women and their families.”

(NFPB p. 15)

FP is framed here as important particularly for the betterment of women’s health with all others who benefit (including their families) centred on women. This reinforces the perception of FP as a matter pertaining to women and reflects the cultural positioning of women as the fulcrum of family structures in Nigeria.

Moreover, women are identified as special targets for FP communication interventions aimed at demand generation:

[On communication strategies] “Specific Demand Generation efforts will be targeted to identify high priority audiences including adolescents, Young people, Married and Unmarried women.”

(NFPCP p. 14)

Here, the gendering of FP is apparent as, besides youths, women—married or not—are singled‐out for special attention; thus suggesting that FP is beneficial for women irrespective of their marital status, but fails to indicate how motivations for using FP or the influencing factors might differ betwixt these two groups of women.

FP is additionally depicted as a means of attaining gender equality in larger society:

[Policy goals include] “Achieving gender equality and elimination of all forms of discrimination through provision of appropriate sexual and reproductive health information and enabling environment for combating sexual coercion, harmful practices and reproductive rights violations”

(NRHP p. 22)

Here, FP is portrayed as being purposely for the prevention of inequitable practices and violence and thus the protection of women who bear the brunt of such acts, alongside the attainment of gender equality overall. Hence, FP is couched as important for the furtherance of women’s rights, although men also benefit immensely from gender equality (King et al., 2018). This framing of FP also implies that a lack of FP access constitutes a way through which women are disadvantaged in larger society, especially in comparison to their male compeers. This presentation of FP also renders it as a *silver bullet* for solving the multifaceted challenge of gender inequality.

Given the aforementioned, the Nigerian government presents FP as the right of women and goes further to hold itself responsible for creating the environment within which they can exercise this right; after which FP becomes the responsibility of women. This is important as gender inequality, the financial, educational and social disempowerment of women and male partner opposition especially (Aji & Omotara, 2018; Alabi et al., 2019; Aransiola et al., 2014; Corroon et al., 2014; Duru et al., 2018), have been variously adduced as impeding women’s FP uptake.

Moreover, in framing FP as meeting the unmet need of women especially, a gendered dimension is apparent and the Nigerian government presents itself as not pushing an agenda it is originating, but responding to its peoples’ wishes. This, nonetheless, appears to overlook the FP wishes of men and inadvertently puts the burden of considering pregnancy prevention and managing the country’s population, on women without saddling men with commensurate responsibility. Consequently, this framing problematises women, leaving them to bear the pregnancy risk while ignoring men’s duty “to ensure that their ejaculations are responsible” during sexual intercourse (Shahvisi, 2020).

Also, this portrayal allows the government to present FP use as having wide support from women, which can then be used to persuade more women to become FP users. The documents also frame FP as important for adolescents—girls in particular—as expounded next.

#### Adolescent girls—Not left out

The Nigerian government signifies that recognition of the rights of adolescents, females especially, to FP, in part, forms the basis for its FP policies. This allows the government to include this demographic, wherein the age of sexual debut lies, in its FP programmes. To this end, the policy reads thus:

“Addressing the sexual and reproductive health needs of Teenage Girls age 10–14 years, particularly the current 10 year old girls and the adolescents age 15–19 years are of critical importance…”

(NRHP p. 14)

As evidenced by this extract, Nigeria pays special attention to females from the age of 10 years, suggesting that they, as compared to their male compeers, face unique challenges in exercising their FP rights. The government also indicates that, in alignment with the age of consent in Nigeria, 11 years (Petroni et al., 2018), it expects adolescent girls to have their sexual debut earlier than boys. Therefore, it makes allowance for the fact that early marriages—particularly for girls who are sometimes married by the point of menarche—are legal and wide‐spread (Amodu et al., 2018),

To facilitate the exercise of adolescents’ right to FP, the education system is presented as a prime target for governmental FP initiatives:

“Family Planning will be incorporated into classroom settings. Teachers will be equipped to adequately support the sexual and reproductive health needs of adolescents and young people”

(NFPCP p. 13)

The Nigerian government, in a bid to meet adolescents where they congregate, focuses on including FP into the educational system. It thus suggests that Nigeria expects adolescents to be in school. That no mention is made of how to target those who might be out of school for different reasons, including conventional ‘marital duties’ like taking care of their husbands/households, buttresses this.

Again, specific strategies are directed at parents also. Case in point, the government aims to:

“…convince parents through Parents Teachers Associations to talk to their children and wards on Family Planning once they start to ask questions on the subject.”

(NFPCP p. 12)

This targeting of parents is apropos as adolescents are typically dependants and under familial guardianship. Nigeria seeks to secure the buy‐in of parents so they enable the exercise of adolescents’ right to FP. This also helps pre‐empt parental opposition to these governmental initiatives. Nonetheless, the government omits any reference to the parental role in facilitating child marriage, implying a reluctance to engage with the issue.

In adopting this rights‐based approach, the Nigerian government presents FP as the entitlement of all adolescents, girls especially and seeks to empower them with the knowledge necessary to access it. Therefore, framing FP as a human right of this demographic provides the grounds for facilitating the exercise of their rights.

#### Men’s right as supporters

As with women, Nigeria couches FP as a right of men. However, men are then framed as needing to support women in exercise of their own right to FP. The following quote represents FP as a men’s right:

“Implicit in this last condition are the right of men… to be informed and to have access to safe, effective, affordable and acceptable methods of family planning of their choice, as well as other methods of their choice for the regulation of fertility which are not against the law”

(NRHP p. 1)

Nigeria characterises FP as the right of men for them to manage their own fertility and choose their fertility patterns. Again, the government puts the responsibility on itself to create the enabling environment for men to exercise this right.

However, in its depictions of men, the government positions them as consequential to the realisation of women’s right to FP. The policies say:

“Also, men's health status and behaviour affect women's SRH status and overall health. Involving men in reproductive health issues has the potential to increase their awareness of SRH issues, improve their health behaviour, as well as increase their acceptance and support to their partners’ needs, choices and rights. Therefore, improving male involvement and participation is key to achieving desired reproductive health outcomes in Nigeria. …Currently, increasing number[s] of programmes and interventions are incorporating specific actions aimed at bringing to the fore male participation in RH issues”

(NRHP p. 13)

Here, men are depicted as not partaking sufficiently in FP generally, strongly influential over women, able to impact women’s FP uptake and as an obstacle to women accessing their right to FP. Portraying men in this manner is congruent with characterisations of men as gatekeepers of women’s health access in the literature, due to gender power imbalances (Aji & Omotara, 2018; Aransiola et al., 2014) and also the patriarchal culture of Nigeria (Okafor et al., 2022; Osezua & Agholor, 2019). Consequently, Nigeria paints men as an important constituency to concentrate efforts on not just to better their own health behaviour, but so as to make them accept and respect their female partners’ FP rights. Such an approach that paints men as decision makers, however, in effect fails to tackle the root of gendered power relations, but may instead uphold the existing patriarchal structures.

## DISCUSSION

This analysis has provided insights into the Nigerian government’s viewpoint on the intersection betwixt gender and family planning (FP). Gender matters feature prominently in the Nigerian government’s outlook on FP and its strategies for promoting FP use. Specifically, Nigeria targets women who are not currently FP users, but have expressed a desire to either restrict or space their childbearing, that is, women with an “unmet need” for FP (FMoH, 2014). This “unmet need” construct presents FP as a matter chiefly within the purview of women’s issues.

Furthermore, Nigeria takes a rights‐based approach to FP promotion, construing FP as a human right. This is in tandem with the United Nations and the literature, which hold that FP enables the right of individuals to control their fertility patterns (Raday et al., 2017; Sinai et al., 2020; UNFPA, 2018). These rights are presented as applying to women and adolescents, females especially, alongside men. Majorly, Nigeria couches FP as a woman’s right that enables her to gain control over her reproductive body and liberally enjoy her sexuality. This further evidences Nigeria’s overwhelming framing of FP as a women’s issue, an approach it continues by giving special focus to adolescent girls. FP as a men’s right is mentioned, albeit briefly, but men are mainly positioned as obstructions to women’s use of FP and thus needing to be converted to supporters through various interventions. This also reflects the gender power imbalance underscored by the prevalent patriarchal culture in the country. Paradoxically, by highlighting men’s position in society and targeting them as decision makers/gatekeepers, Nigeria upholds the existing unequal gender structures (often forcing women to obtain their partners’ approval before accessing FP), instead of tackling the root of the problem—gender inequality and patriarchy.

Notwithstanding, Nigeria also frames FP information as a tool for “achieving gender equality and elimination of all forms of discrimination” (FMoH, 2017b), thus creating the perception that the government wishes to promote women’s rights. However, the policies do not go further to indicate how FP might fit into a holistic strategy for promoting gender equality in general or if such a strategy exists. This constitutes an opportunity for policy convergence in achieving shared gender equality promotion goals across government departments.

Nonetheless, the majority of the recommended interventions are either targeted at women or require them to take action, but an equivalent responsibility is not placed on the men. This indicates a feminisation of FP in Nigeria similar to the findings of Wigginton et al. (2015), who maintain that prevailing discourses of FP paint it as an issue for women to handle. Consequently, this appears to ignore the *male responsibility* in conception and pregnancy (Shahvisi, 2020).

To remedy this, men should be more strongly targeted both for male FP method use and to support their wives or female partners both financially and morally in their FP use, as indeed most FP methods are designed for women (Wigginton et al., 2015). Studies both within Nigeria and in other countries have shown that men‐focussed programmes, for example, FP awareness campaigns and communication programmes targeting men, can be effective in changing men’s attitudes towards FP, gender and SRH issues in general (Aristide et al., 2020; Babalola et al., 2015; Okigbo et al., 2015).

We note a missed opportunity for policy synergy in the fact that the 2021 NPPSD fails to formally build upon the FMoH FP policies (NPC, 2021) despite mentioning FP and fertility management amongst its almost 30 implementation strategies. This could portend a repeat of the ‘weak coordination’ cited as a cardinal reason for the ‘inadequate’ implementation of the preceding 2004 NPPSD (NPC, 2021). A proper evaluation of how the 2021 NPPSD might overlap with/address the issues we raise with the FMoH policies warrants serious assessment in future research, with due attention to the role of the FMoH as a central health and FP service provider.

Hereunder, we expound further crucial points arising from the analysis.

### FP and special women/girls groups

Although unmarried women are mentioned in the policies as high priority intervention targets, there is no indication of if/how their FP needs might differ from married women both in terms of motivation and access. For instance, unmarried women might not have male partners who might hamper their access to FP. Similarly, little research has been conducted focussing on this demographic in Nigeria, who are said to comprise 20% of the unmet need for FP in developing nations (Cleland et al., 2014).

As unmarried women are predominantly young people, a look at the SRH online information preferences of young people in Nigeria and across the world might be instructive. Findings of van Clief and Anemaat (2019) illustrate that young people search more for pleasure‐focussed sexual information online than SRH. Hence, it might be of strategic benefit for the Nigerian government to specially incorporate messaging on sexual pleasure into FP promotion approaches targeting unmarried women. As Laan et al. (2021) posit, sex plays a significant role in relationships, especially with regard to the gratification obtained therefrom. Hence, this approach would be consistent with feminist positionings of FP as enabling female sexual expression by providing control over the possibility of pregnancy (Caruso et al., 2022; Wigginton et al., 2015). Nigeria’s recognition of the right to a satisfying sex life (FMoH, 2017b) already provides the foundation for this approach. This framing of FP as enabling sex for pleasure, as well as providing reproductive control could also be applied to married couples.

Another demographic of concern in the policies is adolescents, with special attention paid to females from 10 years onwards. Nigeria frames FP and reproductive health at large as their human right, which is apropos especially for females, given that child marriage impacts over 40% of Nigerian girls (UNICEF, 2018). There is, however, no mention of child marriage itself or governmental efforts against the practice, but instead the policies seek to support adolescent girls in managing their fertility with FP. This suggests a reluctance by the government to engage in cultural change at best or an endorsement of such marriages at worst.

Furthermore, the policies are unclear about how to specifically target this group of child brides for FP promotion as they are unlikely to be in schools, which are mentioned as the avenue for reaching adolescents. Hence, the government ought to outline ways of including the married out‐of‐school adolescents, look towards challenging cultural factors underpinning early marriages and specifically explore how FP promotion might facilitate the empowerment and protection of the girl‐child.

Nigeria also recognises that it has 50% of the worldwide cases of obstetric fistulae (OF) (FMoH, 2017b), caused by extended childbirth and common in pregnancies of adolescent girls or young women. Missing, however, from the policies is any characterisation of FP as a means of preventing OF, although the UN and the Nigerian strategies on OF prevention highlight FP as one of the key ways of achieving this (FMoH, 2019; UN, 2016). Here lies another opportunity for policy synergy to empower girls and (young) women and secure their (reproductive) health.

### Heterosexual relationships, power and FP

Now considering the imbalance of power within heterosexual relationships (Amiesimaka & Payam, 2020), women have been known to “self‐silence” themselves due to prevalent socialisation that prioritises their families and communities alongside motherhood—which majorly impacts female identity—over women’s personal needs (Logan et al., 2022; Whiley et al., 2021). This self‐silencing is employed as a dispute‐avoidance strategy so the woman avoids negative reactions from the man if she confronts him on a personal, health or sexual issue (Maji & Dixit, 2019). Women also self‐silence and refrain from speaking about their own needs so as to play the “good woman” role, complying with the societal archetypes of the caring and nurturing woman, in many ways to the detriment of their health (Waldron, 2019). This self‐silencing behaviour has further been associated with poor health and social outcomes, such as low condom use and worse depression (Maji & Dixit, 2019; Stokes & Brody, 2019).

These postulations are important for the Nigerian FP context given the preponderance of research showing the gender power imbalance that exists between heterosexual couples (Amiesimaka & Payam, 2020; Osezua & Agholor, 2019). Nigerian women, even though expressing an intention to limit or stop births have been found to demur in bringing it up with their male partners so as not to jeopardise their marital/relationship status given the risk of facing the stigma of being labelled as promiscuous and the risk of destabilising the relationship (Aji & Omotara, 2018; Aransiola et al., 2014; Duru et al., 2018; Mosha et al., 2013).

Considering that there has been limited growth in FP use in the country for around 30 years despite long‐standing and repeated efforts of the Nigerian government in FP promotion (FMoH, 2017a), a change in approach should be considered and this dimension of gender (in)equality focussed on more. In the same vein, FP uptake would benefit if Nigerian women were more empowered to overcome the socio‐cultural norms of childbearing and the “good wife” archetype to prioritise their reproductive rights and bodies. This would enable them to have just as many children as they wish and speak to their husbands/male partners about FP in a non‐permission‐seeking manner.

Consequently, Nigeria should push the message of men and society, respecting women’s rights to choose and control their bodies without fear of stigma or discrimination. To achieve this, a conscious effort is needed in promoting a more egalitarian dynamic and gender equality within marriages/unions and society at large. Hence, men should be a programmatic target for not just getting their buy‐in to “allow” the women use FP, but to promote gender equality so that such permission is not needed.

### FP methods choice and women’s bodily integrity

Nigeria prioritises the promotion of modern (medical/hormonal) methods of FP over natural ones. This represents a medicalisation of FP, as it is approached mainly from a biomedical perspective with medical techniques applied. Hence, all FP options might not be equally available to women folk. These modern methods are overwhelmingly for women’s use, again indicating the gendered nature of FP. Nigeria, nonetheless, acknowledges the right to choose the FP method best suited to the individual. Across studies, some women have expressed a preference for natural methods of FP over medical methods given the side effects of the latter, which natural methods do not have (Alspaugh et al., 2020; Alspaugh et al., 2021; NHS, 2024). More so, the fact that modern methods are administered/implanted/injected into a woman’s body has raised concerns amongst some women of potential infringement on their bodily integrity (Alspaugh et al., 2021)—which is their human right (Raday et al., 2017). This is especially given the disruption of their ‘natural' bodily processes, such as their ‘regular’ menstrual cycle, concomitant with hormonal contraceptives (Alspaugh et al., 2021). Consequently, providing equal access to all methods would be more respectful of women’s right to choose their preferred FP method. The government itself acknowledges this in the policies by encouraging women to keep trying different FP methods until they find the right one for themselves in terms of side effects.

Furthermore, this opportunity to choose is of material importance given the opposition to modern FP methods based on religious grounds, especially within Muslim and Catholic communities (Ataullahjan et al., 2019; Oluwasanu et al., 2019). Therefore, if natural methods are not promoted, this might serve as a barrier to FP uptake. Consequently, a further programmatic step the Nigerian government could take is to ensure the provision of access to *natural* methods, alongside modern medical/hormonal methods as a means of circumventing religious objections to the latter (Barranco & Soler, 2017). It is instructive to note that, with proper application, both are similarly efficacious in preventing pregnancy (WHO, 2023a).

Nonetheless, we acknowledge that modern methods have a better ease‐of‐use than natural methods, as the latter take longer to master (e.g. learning how to monitor one’s menstrual cycle). Again, as natural (fertility awareness) methods require the avoidance of unprotected sex during periods of ovulation, they are difficult to adopt without the male partner’s agreement (Kibira et al., 2020). Thus, women are constrained to employ certain tactics to manoeuvre around the men including: travelling for family visits, feigning illness or instigating conflicts with their husbands in these periods (Izugbara & Ezeh, 2010).

## CONCLUSION

Altogether, the Nigerian government’s policy perspectives on gender and FP align broadly with those shown in the literature (Amiesimaka & Payam, 2020). However, FP uptake unfortunately remains low, with programmatic targets missed; thus suggesting that several factors might be impacting FP uptake and/or the policies may not have been appropriately implemented. Hence, findings and recommendations from this study would be beneficial for the optimisation of the policies.

### Limitations and future research

The findings we present in this study should not be considered gospel as our data analysis was inevitably shaped by our perspectives. Thus, the data could have been interpreted in other ways by other researchers. As with all document analyses, it was not possible to get further data than was available; this was a limitation to this study.

Given the sub‐par FP uptake levels in the country, due consideration should be given to the execution of the plans, focussing on the decision‐makers’ perspectives and also target audiences’ (women, adolescents, etc.) experiences of the implementation of the policies. This would also illuminate deficiencies in the implementation process. More engaging research methods such as interviews or surveys should be used for this purpose. Again, further research could explore the views of women themselves and other FP uptake influencers, such as men, as well as national and sub‐national political leaders who are purse‐holders and thus drive the policy priorities. This would be useful in gaining a balanced view of the impact of gender on FP in Nigeria.

## AUTHOR CONTRIBUTIONS

**Obreniokibo Ibifubara Amiesimaka**: Conceptualization (equal); Data curation (lead); Formal analysis (lead); Investigation (lead); Methodology (equal); Project administration (lead); Resources (lead); Writing—original draft (lead); Writing—review and editing (equal). **Shahin Payam**: Conceptualization (equal); Methodology (equal); Project administration (supporting); Supervision (lead); Writing—review and editing (equal).

## CONFLICT OF INTEREST STATEMENT

The authors declare no conflicts of interest.

## ETHICS STATEMENT

As this study entailed thematic analysis of publicly available documents, specific ethics committee approval was not required.

## PERMISSION TO REPRODUCE MATERIAL FROM OTHER SOURCES

All materials used are cited and referenced accordingly.

## Data Availability

The data that support the findings of this study are available from the corresponding author upon reasonable request.
